# CTGF (IGFBP-rP2) is specifically expressed in malignant lymphoblasts of patients with acute lymphoblastic leukaemia (ALL)

**DOI:** 10.1054/bjoc.2000.1364

**Published:** 2000-08-17

**Authors:** P Vorwerk, H Wex, B Hohmann, Y Oh, R G Rosenfeld, U Mittler

**Affiliations:** Department of Pediatric Hematology and Oncology, Otto von Guericke University Magdeburg, E. -Larisch-Weg 17–19, Magdeburg, D-39112, Germany; Department of Pediatrics, Oregon Health Sciences University, Portland, OR, USA

**Keywords:** CTGF, IGFBP-rP2, mRNA, acute lymphoblastic leukaemia, cell line

## Abstract

Connective tissue growth factor (CTGF) is a major chemotactic and mitogenic factor for connective tissue cells. The amino acid sequence shares an overall 28–38% identity to IGFBPs and contains critical conserved sequences in the amino terminus. It has been demonstrated that human CTGF specifically binds IGFs with low affinity and is considered to be a member of the IGFBP superfamily (IGFBP-rP2). In the present study, the expression of CTGF (IGFBP-rP2) in human leukaemic lymphoblasts from children with acute lymphoblastic leukaemia (ALL) was investigated. RNA samples from tumour clones enriched by ficoll separation of bone marrow or peripheral blood mononuclear cells (MNC) from 107 patients with childhood ALL at diagnosis and 57 adult patients with chronic myeloid leukaemia (CML) were studied by RT-PCR. In addition MNC samples from children with IDDM and cord blood samples from healthy newborns were investigated as control groups. Sixty-one percent of the patients with ALL (65 of 107) were positive for CTGF (IGFBP-rP2) expression. In the control groups, no expression of CTGF (IGFBP-rP2) in peripheral MNC was detected, and in the group of adult CML patients only 3.5% (2 of 57) were positive for this gene. The role of CTGF (IGFBP-rP2) in lymphoblastic leukaemogenesis requires further evaluation, as does its potential utility as a tumour marker. © 2000 Cancer Research Campaign


*British Journal of Cancer* (2000) **83**(6), 756–760
© 2000 Cancer Research Campaign
doi: 10.1054/ bjoc.2000.1364, available online at http://www.idealibrary.com on IDEAL®

### CTGF (IGFBP-rP2) is specifically expressed in malignant lymphoblasts of patients with acute lymphoblastic leukaemia (ALL)

**P Vorwerk[1], H Wex[1], B Hohmann[1], Y Oh[2], RG Rosenfeld[2] and U Mittler[1]**

[1]Otto von Guericke University Magdeburg, Department of Pediatric Hematology and Oncology, E. -Larisch-Weg 17–19, D-39112 Magdeburg, Germany;
[2]Oregon Health Sciences University, Department of Pediatrics, Portland, OR, USA

**Summary** Connective tissue growth factor (CTGF) is a major chemotactic and mitogenic factor for connective tissue cells. The amino acid sequence shares an overall 28–38% identity to IGFBPs and contains critical conserved sequences in the amino terminus. It has been demonstrated that human CTGF specifically binds IGFs with low affinity and is considered to be a member of the IGFBP superfamily (IGFBP-rP2). In the present study, the expression of CTGF (IGFBP-rP2) in human leukaemic lymphoblasts from children with acute lymphoblastic leukaemia (ALL) was investigated. RNA samples from tumour clones enriched by ficoll separation of bone marrow or peripheral blood mononuclear cells (MNC) from 107 patients with childhood ALL at diagnosis and 57 adult patients with chronic myeloid leukaemia (CML) were studied by RT-PCR. In addition MNC samples from children with IDDM and cord blood samples from healthy newborns were investigated as control groups. Sixty-one percent of the patients with ALL (65 of 107) were positive for CTGF (IGFBP-rP2) expression. In the control groups, no expression of CTGF (IGFBP-rP2) in peripheral MNC was detected, and in the group of adult CML patients only 3.5% (2 of 57) were positive for this gene. The role of CTGF (IGFBP-rP2) in lymphoblastic leukaemogenesis requires further evaluation, as does its potential utility as a tumour marker. © 2000 Cancer Research Campaign

**Key words**: CTGF; IGFBP-rP2; mRNA; acute lymphoblastic leukaemia; cell line

CTGF has been cloned from human umbilical vein endothelial cells and identified as a major chemotactic and mitogenic factor for connective tissue cells (Bradham et al, 1991). The protein shares a 28–38% overall amino acid identity to the classical IGFBPs and contains critical conserved sequences, including the common IGFBP motif GCGCCxxC in the N-terminal part. After demonstration of IGF binding to CTGF, it was termed IGFBP-8 (Kim et al, 1997), but, because the IGF binding affinity was very low, IGFBP-8 was renamed insulin-like binding protein-related protein 2 (IGFBP-rP2) (Baxter et al, 1998). Insulin-like growth factors and their binding proteins have been shown to be an integral part of the growth modulation of a number of neoplasms, either by direct interaction with the IGF signalling pathway or in an IGF-independent manner (Oh et al, 1995; Westley and May, 1995; Baserga et al, 1999). CTGF (IGFBP-rP2) is the second of ten identified members of a low-affinity binding subgroup in the IGFBP superfamily, and appears to regulate cell growth through a largely IGF-independent mechanism (Hwa et al, 1999*a*). Nomenclature of this group of molecules has become controversial as investigators from a wide variety of backgrounds have found evidence of the CTGF (IGFBP-rP2) compounds in the course of their studies. The presence of many well-conserved functional domains belonging among others to the CCN and to the IGFBP superfamily and the wide spectrum of biological activities led to multiple name changes that are confusing. Despite the fact that we approached our studies from the IGF field, we are going to use the acronym CTGF (IGFBP-rP2), which at this time may be most appropriate. The CTGF (IGFBP-rP2) gene encodes a 38-kDa prepeptide containing 349 amino acids. Northern blot analysis of various normal human tissues showed expression of CTGF (IGFBP-rP2) mRNA at high levels in spleen, ovary, gastrointestinal tract, prostate, heart and testis and up-regulation of CTGF (IGFBP-rP2) after TGF-β stimulation of human breast cancer cells (Yang et al, 1998; Hishikawa et al, 1999). No expression was found in peripheral blood leukocytes, liver and brain (Kim et al, 1997). CTGF (IGFBP-rP2) was found to be highly expressed in fibrotic skin diseases and negatively correlated with the malignant potency of mesenchymal tumours, as well as their ability to express CD34 antigen (Igarashi et al, 1998). Furthermore, positive expression of CTGF (IGFBP-rP2) was found in the fibrous stroma of mammary tumours and in neuroblastomas and gliomas, although not in all specimens investigated (Frazier and Grotendorst, 1997). Since a number of groups have reported changes in serum or cerebrospinal fluid levels of IGFBPs in patients with ALL, IGFBPs and their related proteins have became subjects of intensive research in human neoplasia (Muller et al, 1994; Mohnike et al, 1996; Kim et al, 1997; Wex et al, 1998; How et al, 1999).

In the present study, we have investigated the expression of CTGF (IGFBP-rP2) in leukaemic cells of patients with ALL, or in established leukaemia cell lines, by RT-PCR. Leukaemic cells from patients with CML, peripheral MNC from patients with IDDM, $CD34^+$ stem cells from cord blood of healthy newborns and peripheral $CD34^+$ stem cells from patients with sarcomas were used as controls.

*Received 7 January 2000*
*Revised 19 April 2000*
*Accepted 8 June 2000*

*Correspondence to:* P Vorwerk

### MATERIALS AND METHODS

### Patient samples

Blood or bone marrow samples from 107 patients (age 1.2–21.9 years, median 6.0 years) with childhood acute lymphoblastic leukaemia (ALL) at time of diagnosis (B-precursor ALL, *n* = 15; B-ALL, *n* = 2; T-ALL, *n* = 22; c-ALL, *n* = 67; pre-T-ALL, *n* = 1) from different ALL-BFM study centres were obtained. None of the ALL-patients received any drugs before the first blood or bone marrow sample was drawn. From 43 of these patients (B-precursor ALL, *n* = 6; B-ALL, *n* = 2; T-ALL, *n* = 8; c-ALL, *n* = 27), additional samples were obtained at day 33 after the initiation of chemotherapy, according to the ALL-BFM 90/95 protocol of the German Society of Pediatric Oncology and Hematology.

All 43 patients were in full haematological remission at day 33. Additionally, blood or bone marrow samples from 57 patients (age 6.4–84 years, median 47.8 years) with chronic myeloid leukemia (CML), 120 blood samples from 47 children (age 1.8–15.8 years, median 10.9) with insulin-dependent diabetes mellitus (IDDM), 100 cord blood samples from healthy newborns, and two samples of peripheral stem cells from patients with sarcomas, before high-dose chemotherapy for stem-cell rescue, were investigated (Tables 1 and 2). Informed consent was obtained from the participating patients or their parents in adherence with the guidelines of the Ethics Committee. The cytometric immunophenotyping of haematopoietic malignancies was carried out in local and reference laboratories according to a consensus protocol (Rothe and Schmitz, 1996).

### Cell lines and cultures

Cell lines were obtained from the German Collection of Microorganisms and Cell Cultures (Braunschweig, Germany) or ATCC (Rockville, MD, USA) and grown according to the recommended conditions. Molt-4, Karpas 299, Jurkat and HSB-2 are of T-cell origin, whereas Karpas 422, cell line 380 and cell line 697 are of B-cell origin (Table 3). The origins of Raji, JVM-3, JVM-13 and U 937 are specified in Table 3.

### Separation of mononuclear cells (MNC)

MNC were separated using gradient centrifugation with Ficoll-Paque (Pharmacia, Uppsala, Sweden). At the time of diagnosis, the tumour clones generally represented 50–90% of MNC fraction.

### Separation of $CD34^+$/$CD38^+$ stem cells

$CD34^+$/$CD38^+$ progenitor cells were stained from peripheral blood and separated by standard methods, using a FACSort machine (Becton Dickinson, Heidelberg, Germany). Approximately 100 000 cells were separated and used for extraction of RNA.

### RNA isolation/cDNA synthesis

Total RNA was extracted using QIAshredder®, RNeasy®, and RNase-free DNase set (Qiagen, Hilden, Germany), following the manufacturer's protocol. The RNA was stored at –80°C until use. 2 μg of total RNA were reverse transcribed into cDNA by Omniscript reverse transcriptase (Qiagen), as recommended by the manufacturer.

### Enzymatic amplification of the cDNA

0.5 μl aliquots of cDNA were used for enzymatic amplification in 50 μl reactions containing 10 × reaction buffer mixture, 1 unit Prime Zyme Taq-polymerase (Biometra, Göttingen, Germany) and

**Table 1** Expression of CTGF (IGFBP-rP2) in MNC of patients with ALL, CML, IDDM and sarcoma as well as cord blood of healthy newborns. 60.7% of all ALL patients were positive for CTGF (IGFBP-rP2) expression in MNC. [a]There is a statistically significant difference in CTGF (IGFBP-rP2) expression in the malignant lymphoblasts between the immunological subtypes of ALL. Only 1.5% of CTGF (IGFBP-rP2)-positive patients showed a T-immunophenotype, whereas 52% of negative patients have been classified as T-ALL. [b]The control groups differ significantly from the ALL group in regard to CTGF (IGFBP-rP2) expression. Only CML patients expressed in 3.5% CTGF (IGFBP-rP2). IDDM patients and isolated $CD34^+$ stem cells were CTGF (IGFBP-rP2)-negative

| Patients | Material | Cell type | *n* = | CTGF expression | Origin |
|---|---|---|---|---|---|
| ALL[b] | Blood or bone marrow | MNC | 107 | positive* 60.7% (*n* = 65) | B-cell: 98.5% (*n* = 64) T-cell: 1.5% (*n* = 1) |
| | | | | negative* 39.3% (*n* = 42) | B-cell: 47.6% (*n* = 20) T-cell: 52.4% (*n* = 22) |
| CML[b] | Blood or bone marrow | MNC | 57 | positive 3.5% (*n* = 2) | |
| | | | | negative 96.5% (*n* = 55) | |
| IDDM[b] | Blood | MNC | 120 | negative 100% (*n* = 120) | |
| Sarcoma | Blood | CD $34^+$ | 2 | negative 100%(*n* = 2) | |
| Newborns[b] | Cord blood | CD $34^+$ | 100 | negative 100% (*n* = 100) | |

*© 2000 Cancer Research Campaign*

**Table 2** Expression of CTGF (IGFBP-rP2) in 43 patients with ALL at different time-points. Expression in lymphoblasts was studied by RT-PCR at diagnosis and at day 33 according the ALL-BFM-therapy protocol. The values are expressed in percent of the respective group with the 95% confidence interval

| CTGF at diagnosis (total *n* = 43) | CTGF at day 33 (total *n* = 43) | | | In % of total *n* |
|---|---|---|---|---|
| | | Negative | 83.3% (62.6–95.3%) (*n* = 20) | 46.5% |
| Positive | 55.8% (*n* = 24) | | | |
| | | Positive | 16.7% (4.7–37.4%) (*n* = 14) | 9.3% |
| | | Negative | 73.7% (48.8–90.8%) (*n* = 14) | 32.6% |
| Negative | 44.2% (*n* = 19) | | | |
| | | Positive | 26.3% (9.2–51.2%) (*n* = 5) | 11.6% |

**Table 3** Expression of CTGF (IGFBP-rP2) mRNA by RT-PCR in different lymphatic cell lines. Only cell lines established from patients with childhood ALL of B-origin (380, 697) were found to be positive for CTGF (IGFBP-rP2) expression

| Cell line | Origin | CTGF (IGFBP-rP2) |
|---|---|---|
| Raji | Established from the left maxilla of an 11-year-old African boy with Burkitt's lymphoma | Negative |
| JVM-3 | Human chronic B cell leukaemia, established from the peripheral blood of a 73-year-old man with B-prolymphocytic leukaemia | Negative |
| JVM-13 | Human chronic B cell leukaemia, established from the peripheral blood of a patient with B-prolymphocytic leukaemia | Negative |
| Karpas 422 | Pleural effusion of a 73-year-old woman with B cell Non-Hodgkin's lymphoma | Negative |
| U 937 | Pleural effusion of a 37-year-old man with histiocytic lymphoma cells express markers and properties of monocytes | Negative |
| Molt-4 | Peripheral blood of a 19-year-old man with T-ALL in relapse | Negative |
| Karpas 299 | Peripheral blood of a 25-year-old man with T cell non-Hodgkin's lymphoma | Negative |
| Jurkat | Peripheral blood of a 14-year-old boy with T-ALL | Negative |
| HSB-2 | Peripheral blood of an 11.5-year-old boy with T-ALL | Negative |
| 380 | Peripheral blood of a 15-year-old boy with B-ALL in relapse | Positive |
| 697 | Bone marrow of a 12-year-old boy with c-ALL at relapse | Positive |

0.2 pmol of both gene-specific primers in a Hybaid Gene Thermocycler (Hybaid). Initial denaturation at 95°C for 5 min was followed by 35 cycles with denaturation at 95°C for 1 min, annealing at 62°C for 1 min and elongation at 72°C for 0.5 min. The final elongation step was extended to 15 min. One-fifth of the reaction mix was loaded onto a 1.75% agarose gel, separated by electrophoresis at 5 V $cm^{-1}$ in TAE buffer and stained with ethidium bromide. CTGF forward primer: CAACTGCCTG-GTCCAGACC corresponding to nucleotide numbers 371–389; CTGF reverse primer: CACTCTCTGGCTTCATGCC corresponding to nucleotide numbers 842–824 of the human CTGF-mRNA, GenBank accession number U14750. β-actin forward primer: GCTGGGGTGTTGAAGGTCTC; β-actin reverse primer: CTCCGGCATGTGCAAGGC.

The resulting PCR products are 471 bp (CTGF) and 351 bp (β-actin) long (Figure 1).

### Statistical analysis

For statistical analysis $\chi^2$ test was performed with SPSS Version 9.1 at $P = 0.001$. Confidence intervals for 95% are estimated according to the number of patients using confidence interval tables.

### RESULTS

MNC from 60.7% of the patients with ALL (65 of 107) were positive for CTGF (IGFBP-rP2) expression at diagnosis. All other groups are statistically significantly different from the ALL group ($P < 0.001$). In the group of CML patients, MNC from only 3.5% (2 of 57) were positive for CTGF (IGFBP-rP2). No expression was detected by RT-PCR in MNC from diabetics, cord blood samples or peripheral stem cells (Table 1).

98.5% ($n = 64$) of the patients in the group of CTGF (IGFBP-rP2) expression positive ALL patients ($n = 65$) showed an immunological subtype of B-cell origin (c-ALL or B-ALL) and only 1–5% ($n = 1$) was from T-cell origin. The CTGF (IGFBP-rP2) negative ALL patients are statistically significant ($P < 0.001$) different in their immunological background. 52.4% ($n = 22$) of CTGF (IGFBP-rP2) negative ALL patients were immunologically classified as T-ALL and only 47.6% ($n = 20$) were immunologically from B-cell origin (Table 1).

© 2000 Cancer Research Campaign

We were able to investigate a second MNC sample from day 33 of chemotherapy in 43 ALL patients. On this day, all 43 patients had achieved haematological remission. Twenty-four (55.8%) of these 43 ALL patients expressed CTGF (IGFBP-rP2) mRNA at diagnosis. Twenty patients (83.3%) of these 24 initially CTGF (IGFBP-rP2)-positive patients became negative for CTGF (IGFBP-rP2) after the initial treatment, whereas four patients remained positive (16.7%). From the 19 of 43 (44.2%) initially CTGF (IGFBP-rP2)-negative ALL patients, 14 remained negative and five became positive for CTGF (IGFBP-rP2) mRNA expression (Table 2).

From the 11 cell lines studied for CTGF (IGFBP-rP2) expression only those established from patients with childhood ALL of B-cell origin (380 and 697) were found to express CTGF (IGFBP-rP2) mRNA by RT-PCR (Table 3).

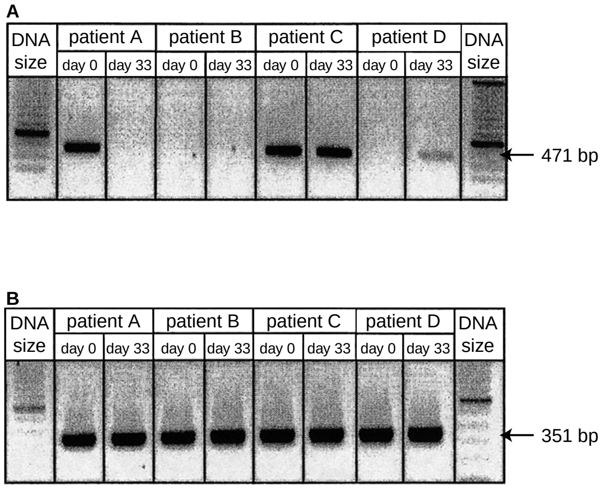

**Figure 1** CTGF (IGFBP-rP2) expression in four different patients at diagnosis and remission (day 33) according to the ALL-BFM90/95 therapy study protocol (upper panel). The lower panel shows the β-actin controls of the cDNA used for RT-PCR.

### DISCUSSION

Insulin-like growth factor binding proteins (IGFBPs) are a group of homologous proteins that regulate the biological activities of IGFs and may also act in an IGF-independent manner (Oh et al, 1993; Rosenfeld et al, 1994; Jones and Clemmons, 1995). In addition to the six well-characterized IGFBPs with high affinity for IGF, at least ten additional proteins have been reported to have a significant structural relationship (Hwa et al, 1999*a*; 1999*b*). These proteins contain conserved cysteines in the $NH_2$-terminal region, including the ‘IGFBP-motif’ (GCGCCXXC). To date, specific IGF binding has been described in at least three new members of the IGFBP superfamily, MAC25 (IGFBP-rP1), CTGF (IGFBP-rP2), and Nov-H (IGFBP-rP3) (Oh et al, 1996; Kim et al, 1997; Burren et al, 1999).

Proceeding from our previous reports on expression of insulin-like growth factors and their binding proteins in human leukaemic lymphoblasts, we studied the gene expression of new members of the IGFBP superfamily in leukaemia (Wex et al, 1998). Preliminary RT-PCR data suggested, that in contrast to Mac25 (IGFBP-rP1), Nov-H (IGFBP-rP3) and Cyr61 (IGFBP-rP4), only CTGF (IGFBP-rP2) is specifically expressed in leukaemic lymphoblasts (data not shown).

In the present study we demonstrate by RT-PCR that CTGF (IGFBP-rP2) mRNA is specifically expressed in more than 60% of patients with acute lymphoblastic leukaemia at the time of diagnosis. In contrast to this finding, no expression of CTGF (IGFBP-rP2) mRNA could be detected in peripheral blood lymphocytes of control persons with IDDM, myeloid leukaemic cells of CML-patients or stem cells from peripheral blood or cord blood.

Our data demonstrate for the first time that especially leukaemic B-, but also T-cells of patients with ALL are able to express CTGF (IGFBP-rP2). The disappearance of gene expression after chemotherapy in the majority of initial CTGF (IGFBP-rP2)-positive patients may reflect the disappearance of the leukaemia cells themselves. However, although all patients reached apparently complete haematological remission at day 33, 16.7% of all initial CTGF (IGFBP-rP2)-positive patients remained positive, and a number of initially negative patients expressed the gene at this time-point.

To investigate whether the ability of the leukaemic cell population to express CTGF (IGFBP-rP2) is a property of all lymphoid progenitors, and not exclusive for leukaemia cells, we investigated $CD34^+/CD38^+$ cell populations for expression of CTGF (IGFBP-rP2). Our data demonstrate that normal lymphoid progenitor cells from cord blood do not express CTGF (IGFBP-rP2). Furthermore, $CD34^+/CD38^+$ progenitor cells for stem cell rescue from two patients with sarcomas prior to high-dose chemotherapy were negative for CTGF (IGFBP-rP2) expression. Therefore, we conclude, that CTGF (IGFBP-rP2) expression in leukaemic lymphoblasts of patients with childhood ALL is specific.

The fact that only cell lines with B-origin from patients with childhood ALL expressed CTGF (IGFBP-rP2) reflects the situation in our ALL patients. The majority of patients with T-ALL do not express CTGF (IGFBP-rP2) in their malignant lymphoblasts.

At this time, there are insufficient data concerning the outcome of ALL in patient groups with different CTGF (IGFBP-rP2) expression patterns. Further investigations of CTGF (IGFBP-rP2) in ALL and other neoplasms are necessary to investigate the role of this protein in the pathogenesis of these diseases, and its potential role as a tumour marker or MRD parameter.

### ACKNOWLEDGEMENTS

This work was supported in part by a grant from ‘Deutsche Leukämie Forschungshilfe eV.’, by the ‘Magdeburger Förderkreis krebskranker Kinder eV.’, the ‘W. A. Drenckmann – Foundation’, NIH grant DK51513 (RGR), NCI grant CA58110 (RGR) and a grant from the American Cancer Society RPG 99-103-01-TBE (YO).

### REFERENCES

Baserga R, Prisco M and Hongo A (1999) IGFs and cell growth. In *The IGF System; Molecular Biology, Physiology, and Clinical Applications*, RG Rosenfeld and CTJ Roberts (eds), pp 329–353. Humana Press: Totowa, New Jersey

Baxter RC, Binoux MA, Clemmons DR, Conover CA, Drop SL, Holly JM, Mohan S, Oh Y and Rosenfeld RG (1998) Recommendations for nomenclature of the insulin-like growth factor binding protein superfamily. *Endocrinology* **139**: 4036–4036

*© 2000 Cancer Research Campaign* 

Bradham DM, Igarashi A, Potter RL and Grotendorst GR (1991) Connective tissue growth factor: a cysteine-rich mitogen secreted by human vascular endothelial cells is related to the SRC-induced immediate early gene product CEF-10. *J Cell Biol* **114**: 1285–1294

Burren CP, Wilson EM, Hwa V, Oh Y and Rosenfeld RG (1999) Binding properties and distribution of insulin-like growth factor binding protein-related protein 3 (IGFBP-rP3/NovH), an additional member of the IGFBP Superfamily. *J Clin Endocrinol Metab* **84**: 1096–1103

Frazier KS and Grotendorst GR (1997) Expression of connective tissue growth factor mRNA in the fibrous stroma of mammary tumours. *Int J Biochem Cell Biol* **29**: 153–161

Hishikawa K, Oemar BS, Tanner FC, Nakaki T, Luscher TF and Fujii T (1999) Connective tissue growth factor induces apoptosis in human breast cancer cell line MCF-7. *J Biol Chem* **274**: 37461–37466

How HK, Yeoh A, Quah TC, Oh Y, Rosenfeld RG and Lee KO (1999) Insulin-like growth factor binding proteins (IGFBPs) and IGFBP-related protein 1-levels in cerebrospinal fluid of children with acute lymphoblastic leukemia. *J Clin Endocrinol Metab* **84**: 1283–1287

Hwa V, Oh, Y and Rosenfeld RG (1999*a*) Insulin-like growth factor binding proteins: a proposed superfamily. *Acta Paediatr Suppl* **88**: 37–45

Hwa V, Oh Y and Rosenfeld RG (1999*b*) The insulin-like growth factor-binding protein (IGFBP) superfamily. *Endocr Rev* **20**: 761–787

Igarashi A, Hayashi N, Nashiro K and Takehara K (1998) Differential expression of connective tissue growth factor gene in cutaneous fibrohistiocytic and vascular tumours. *J Cutan Pathol* **25**: 143–148

Jones JI and Clemmons DR (1995) Insulin-like growth factors and their binding proteins: biological actions. *Endocr Rev* **16**: 3–34

Kim HS, Nagalla SR, O Y, Wilson E, Roberts CT, Jr. and Rosenfeld RG (1997) Identification of a family of low-affinity insulin-like growth factor binding proteins (IGFBPs): characterization of connective tissue growth factor as a member of the IGFBP superfamily. *Proc Natl Acad Sci USA* **94**: 12981–12986

Mohnike K, Kluba U, Mittler U, Aumann V, Vorwerk P and Blum W (1996) Serum levels of insulin-like growth factor-I, -II and insulin-like growth factor binding protein-2 and -3 in children with acute lymphoblastic leukemia. *Eur J Pediatr* **155**: 81–86

Muller HL, Oh Y, Gargosky SE, Wilson KF, Lehrnbecher T and Rosenfeld RG (1994) Insulin-like growth factor binding protein-3 concentrations and insulin-like growth factor binding protein-3 protease activity in sera of patients with malignant solid tumours or leukemia. *Pediatr Res* **35**: 720–724

Oh Y, Muller HL, Lamson G and Rosenfeld RG (1993) Insulin-like growth factor (IGF)-independent action of IGF-binding protein-3 in Hs578T human breast cancer cells. Cell surface binding and growth inhibition. *J Biol Chem* **268**: 14964–14971

Oh Y, Gucev Z, Ng L, Muller HL and Rosenfeld RG (1995) Antiproliferative actions of insulin-like growth factor binding protein (IGFBP)-3 in human breast cancer cells. *Prog Growth Factor Res* **6**: 503–512

Oh Y, Nagalla SR, Yamanaka Y, Kim HS, Wilson E and Rosenfeld RG (1996) Synthesis and characterization of insulin-like growth factor-binding protein (IGFBP)-7. Recombinant human mac25 protein specifically binds IGF-I and -II. *J Biol Chem* **271**: 30322–30325

Rosenfeld RG, Pham H, Cohen P, Fielder P, Gargosky SE, Muller H, Nonoshita L and Oh Y (1994) Insulin-like growth factor binding proteins and their regulation. *Acta Paediatr Suppl* **399**: 154–158

Rothe G and Schmitz G (1996) Consensus protocol for the flow cytometric immunophenotyping of hematopoietic malignancies. Working Group on Flow Cytometry and Image Analysis. *Leukemia* **10**: 877–895

Westley BR and May FE (1995) Insulin-like growth factors: the unrecognised oncogenes. *Br J Cancer* **72**: 1065–1066

Wex H, Vorwerk P, Mohnike K, Bretschneider D, Kluba U, Aumann V, Blum WF and Mittler U (1998) Elevated serum levels of IGFBP-2 found in children suffering from acute leukaemia is accompanied by the occurrence of IGFBP-2 mRNA in the tumour clone. *Br J Cancer* **78**: 515–520

Yang DH, Kim HS, Wilson EM, Rosenfeld RG and Oh Y (1998) Identification of glycosylated 38-kDa connective tissue growth factor (IGFBP-related protein 2) and proteolytic fragments in human biological fluids, and up-regulation of IGFBP-rP2 expression by TGF-beta in Hs578T human breast cancer cells. *J Clin Endocrinol Metab* **83**: 2593–2596

*© 2000 Cancer Research Campaign*
